# Data to identify key drivers of animal growth and carcass quality for temperate lowland sheep production systems

**DOI:** 10.1016/j.dib.2021.106977

**Published:** 2021-03-18

**Authors:** A.G. Jones, H. Fleming, B.A. Griffith, T. Takahashi, M.R.F. Lee, P. Harris

**Affiliations:** a Rothamsted Research, North Wyke, Okehampton, Devon EX20 2SB, UK; b University of Bristol, Bristol Veterinary School, Langford, Somerset BS40 5DU, UK; c Harper Adams University, Newport, Shropshire TF10 8NB, UK

**Keywords:** Farm management, Grazing livestock, Liveweight, Condition score, Conformation score, Fat class, Lamb, Ewe

## Abstract

With the growing demand for animal-sourced foods and a serious concern over climate impacts associated with livestock farming, the sheep industry worldwide faces the formidable challenge of increasing the overall product supply while improving its resource use efficiency. As an evidence base for research to identify key drivers behind animal growth and carcass quality, longitudinal matched data of 741 ewes and 2978 lambs were collected at the North Wyke Farm Platform, a farm-scale grazing trial in Devon, UK, between 2011 and 2019. A subset of these data was subsequently analysed in a study to assess the feasibility of using a lamb's early-life liveweight as a predictor of carcass quality [1]. The data also have the potential to offer insight into key performance indicators (KPIs) for the sheep industry, or what variables farmers should measure and target to increase profitability.

## Specifications Table

**Table:** 

Subject	Agricultural and Biological Sciences
Specific subject area	Livestock science
Type of data	Table
How data were acquired	On a research farm
Data format	Raw
Parameters for data collection	A research farm operating under a representative production environment for temperate lowland regions
Description of data collection	Body condition of ewes and growth of lambs were both directly measured on the farm. Information on carcass quality was obtained from the abattoir following the slaughter of lambs.
Data source location	Okehampton, Devon, UK (50°46′10″N, 3°54′05″W)
Data accessibility	Repository: Mendeley Data doi: 10.17632/xy3ndcy8jd.1
Related research article	A.G. Jones, T. Takahashi, H. Fleming, B.A. Griffith, P. Harris, M.R.F. Lee, Using a lamb's early-life liveweight as a predictor of carcass quality, Animal 15 (2021) 100018. https://doi.org/10.1016/j.animal.2020.100018.

## Value of the Data

• Longitudinal data were obtained from ewes and their lambs, providing a rare opportunity to decompose a lamb's performance into genetic and non-genetic factors.
• Livestock farming communities and wider society can both benefit from these data, through enhanced profitability and reduced environmental impacts of agriculture, respectively.
• The data can potentially be reused to identify key performance indicators (KPIs) for the sheep industry, guiding farmers what variables they should measure on the farm.

## 1 Data Description

With the growing demand for animal-sourced foods and a serious concern over climate impacts associated with livestock farming, the sheep industry worldwide faces the formidable challenge of increasing the overall product supply while improving its operational and environmental efficiencies [2], [3]. The data presented here were collected from the North Wyke Farm Platform (NWFP) [4], a farm-scale grazing trial in Devon, UK, to assist identification of key drivers behind animal growth and carcass quality within the context of temperate lowland sheep production systems. The data encompass 2978 lambs and their mothers (741 ewes) that belonged to the NWFP over a 9-year period between 2011 and 2019.

All data are publicly available from a data repository [5]. The data take a ‘rectangular’ format with a lamb as the unit of observation, with corresponding ewe information appended to each lamb. This means that an identical set of ewe information appears twice for twin lambs. The following variables are included in the data for each lamb:

• animal ID
• year of production
• sward management (see Section 2)
• date of birth
• date of slaughter
• litter size
• liveweight: date and value
• cold carcass weight
• conformation score
• fat class
• carcass price
• mother's ID
• mother's liveweight at key dates (tupping, lambing and weaning)
• mother's condition score at key dates (tupping, lambing and weaning)

As a case exemplar to demonstrate the value of the data, a subset was subsequently analysed in a study to assess the feasibility of using a lamb's early-life liveweight as a predictor of carcass quality [1].

## 2 Experimental Design, Materials and Methods

The NWFP (50°46′10″N, 3°54′05″W) consists of three self-contained grazing livestock enterprises (21 ha each), which operate under different sward management strategies of reseeded grass monoculture, reseeded legume/grass mix and no reseeding (permanent pasture) [4]. General information on the platform (including an introductory video) is available on a dedicated portal [6]. The NWFP's overall design philosophy [7], environmental appraisal [8] and cattle operation [9] have been discussed as part of separate studies.

The NWFP's past and present sheep operations are also detailed elsewhere [10]. Currently, lambs are produced by a mixed age flock of Suffolk x Mule ewes, mated to Charollais sires in October and November each year. Ewes are housed from December, give births in March and April, and turn out to pasture with lambs at 72 h post-lambing. Under a lambing rate of 1.83, lambs are reared as either singles or twins, with one of the triplet-born lambs either cross-fostered onto a single-rearing ewe or artificially reared with milk replacer. In order to minimise the statistical confoundment attributable to the use of milk replacer, the latter group is immediately excluded from the trial. Lambs are weaned at 13 weeks of age and finished at ~45 kg, typically around October.

The liveweight of lambs was recorded at birth, four weeks, eight weeks, 13 weeks (weaning) and every two weeks thereafter until finishing. For four-week and eight-week weights that are particularly time-sensitive, a linear adjustment was made to estimate the corresponding weight (when measurements were not taken on the exact day) to ensure inter-animal comparability. Cold carcass weight, conformation score, fat class and carcass price for each lamb were obtained from the abattoir following the slaughter. For ewes, the liveweight and condition score [11] were recorded at tupping, lambing and weaning. Both lambs and ewes were weighed individually on a weigh crate. Condition scores for ewes were manually assessed by a trained operator.

## Ethics Statement

All animal data used in this study were collected as part of standard farming practices. As such, no part of this research was subject to approval of an ethics committee.

## CRediT Author Statement

**T. Takahashi, M.R.F. Lee** and **P. Harris:** designed the study; **P. Harris:** oversaw the data collection; **A.G. Jones, H. Fleming** and **B.A. Griffith:** collected the data; **A.G. Jones** and **T. Takahashi:** collated the data; **A.G. Jones** and **T. Takahashi:** prepared the draft. All authors critically reviewed the draft and contributed to the final version of the manuscript.

## Declaration of Competing Interest

The authors declare that they have no known competing financial interests or personal relationships that could have appeared to influence the work reported in this article.
